# Cuspidatyl Ferulate, a Novel Phenolic Acid from *Hyssopus cuspidatus* Borris, Protects Hepatocytes Against Oxidative Damage via Keap1 Interaction

**DOI:** 10.3390/antiox14121449

**Published:** 2025-12-01

**Authors:** Xingyu Liu, Zhao Zhang, Denghui Gao, Xiaoguang Yang, Lei Liu, Guannan Wang, Zhenbo Song, Weiwei Fang, Shuyue Wang

**Affiliations:** 1 National Engineering Laboratory for Druggable Gene and Protein Screening, Northeast Normal University, Changchun 130117, China; liux56698@gmail.com (X.L.); zhangz777@nenu.edu.cn (Z.Z.); songzb484@nenu.edu.cn (Z.S.); 2 School of Life Sciences, Northeast Normal University, Changchun 130032, China; liul905@nenu.edu.cn (L.L.); wanggn258@nenu.edu.cn (G.W.); 3 NMPA Key Laboratory for Quality Control of Cell and Gene Therapy Medicine Products, Northeast Normal University, Changchun 130032, China; gaodh@nenu.edu.cn (D.G.); yangxg168@nenu.edu.cn (X.Y.); 4 Department of Blood Transfusion, State Key Laboratory of Molecular Oncology, National Cancer Center/National Clinical Research Center for Cancer/Cancer Hospital, Chinese Academy of Medical Sciences and Peking Union Medical College, Beijing 100021, China

**Keywords:** antioxidant, *Hyssopus cuspidatus* Borris, Keap1-Nrf2 pathway, phenolic acid, lipotoxicity, MASLD, oxidative stress

## Abstract

Lipotoxicity and oxidative stress are key pathogenic drivers in the development of Metabolic Dysfunction-Associated Steatotic Liver Disease (MASLD). The underlying mechanisms of MASLD are not fully understood, and approved pharmacotherapies remain elusive. Thus, exploring therapeutic targets and potential drugs for MASLD is still a major challenge. In our previous study, a new cuspidatyl ferulate (2,3-dihydroxy-4-carboxylic butyl (E)-4-[3-(4-hydroxy-3-methoxyphenyl)-2-propenoate], CuF) was first isolated and identified from *Hyssopus cuspidatus* Boriss (*H. cuspidatus*). Here, we investigated the effects of this novel phenolic acid on free fatty acid (FFA)-induced oxidative stress and lipid accumulation in HepG2 cells. Exposure to FFA significantly increased intracellular reactive oxygen species (ROS) levels and lipid accumulation. Notably, CuF treatment effectively reversed FFA-induced suppression of key antioxidant enzymes, including superoxide dismutase (SOD) and catalase (CAT), and attenuated lipid accumulation, as evidenced by reduced total cholesterol (TC) and triglyceride (TG) levels. Mechanistically, molecular docking and capillary electrophoresis analyses revealed that CuF directly interacts with Kelch-like ECH-associated protein 1 (Keap1), disrupting the Keap1-Nrf2 protein complex, thereby promoting nuclear translocation of Nrf2 and activating the antioxidant response pathway. In summary, our findings demonstrate that this novel phenolic acid exhibits strong antioxidant and anti-lipotoxic activities *in vitro*, offering a potential natural product-based drug candidate for MASLD therapy.

## 1. Introduction

Metabolic Dysfunction-Associated Steatotic Liver Disease (MASLD), formerly known as nonalcoholic fatty liver disease (NAFLD), is the most common chronic liver disease. It affects 20–30% of the adult population in developed countries, highlighting the increasing burden of metabolic dysfunction on public health [1]. MASLD encompasses a spectrum of hepatic conditions, ranging from isolated steatosis, defined as lipid accumulation in more than 5% of hepatocyte to metabolic dysfunction-associated steatohepatitis (MASH) [2]. However, the pathogenesis of MASLD is complex, and approved medications for its treatment remain limited. Therefore, it is a major challenge to investigate the mechanisms underlying MASLD development and to identify potential therapeutic targets and effective drugs.

The development of MASLD is closely linked to free fatty acid (FFA)-induced lipotoxicity, which results from excessive carbohydrate intake (especially fructose) or from remnant lipoproteins and lipolysis in adipose tissue, subsequently transported to the liver via various uptake transporters [3]. The storage of excess FFA in hepatocytes drives reactive oxygen species (ROS) production, resulting in lipotoxic oxidative damage. This damage propagates a pathological cascade of inflammation and apoptosis, promotes severe liver injury and ultimately progresses to fibrosis [4,5]. Therefore, alleviating FFA-induced oxidative stress represents a pressing challenge in MASLD prevention and treatment [3].

Oxidative stress arises from an imbalance between the generation and removal of ROS. Under normal physiological processes, ROS signaling involved in cell metabolism, survival, proliferation, and immune defense through the modulation of transcription factors and epigenetic pathways [6]. Persistent ROS can cause oxidative modification to cellular macromolecules, such as lipids, proteins, and DNA, ultimately disrupting the structural and functional integrity of cells and inducing cell injury [7]. Oxidative stress can also activate an inflammatory signaling cascade and induce overexpression of many pro-inflammatory factors, thereby exacerbating cellular inflammation.

Cells possess complex intracellular antioxidant defense systems to counteract oxidative stress, and Nrf2 plays a central role in regulating antioxidant signaling. Under steady-state conditions, Nrf2 binds to Keap1 in the cytoplasm; under stress conditions, Nrf2 dissociates from Keap1 and translocates to the nucleus, where it binds to the antioxidant response element (ARE) and promotes transcriptional activation of phase II metabolic enzymes and antioxidant proteases such as catalase (CAT), glutathione peroxidase (GSH-Px), and superoxide dismutase (SOD) [8]. Thus, the NRF2/KEAP1/ARE pathway is a key regulatory system that counters cellular oxidative stress by activating phase II metabolic enzymes and antioxidant proteases.

Growing evidence indicates that polyphenol-rich extracts or isolated polyphenolic monomers, mainly derived from plants, can exert beneficial effects on metabolic disorders by improving metabolism, inhibiting oxidative stress and inflammation, and enhancing insulin sensitivity [9,10]. Natural polyphenols are secondary metabolites of plants and possess notable antioxidant capacity, attributed to their hydroxylated aromatic rings. This structure allows them to scavenge free radicals via proton/electron donation and metal chelation, thereby alleviating oxidative stress and supporting cellular redox balance. Additionally, their dietary availability, efficient absorption, and favorable safety profile enhance their therapeutic potential [11].

*Hyssopus cuspidatus* Borris (*H. cuspidatus*) is an aromatic plant used for both medicinal and food purposes [12,13,14]. As first reported by our group [15], CuF (**cu**spidatyl **f**erulate, 2,3-dihydroxy-4-carboxylic butyl (E)-4-[3-(4-hydroxy-3-methoxyphenyl)-2-propenoate]) is a new phenolic acid isolated from *H. cuspidatus*. In the present study, we further investigated the antioxidant activity of CuF in FFA-induced HepG2 cells. Our results revealed that CuF targets Nrf2/keap1 pathway, thereby reducing oxidative stress and lipid accumulation *in vitro*. These findings highlight the potential of CuF in oxidative stress-related diseases and provide a material basis and theoretical rationale for the application of this natural compound in the prevention and treatment of oxidative damage-related conditions.

## 2. Materials and Methods

### 2.1. Cell Culture and Treatment

Human HepG2 cells were obtained from the Cell Bank of the Chinese Academy of Sciences (Shanghai, China), and THLE-2 cells were purchased from Zhejiang MEISEN Cell Technology Co., Ltd. (Hangzhou, Zhejiang, China). HepG2 cells were cultured in high-glucose DMEM (Thermo Fisher Scientific, Waltham, MA, USA) supplemented with 10% fetal bovine serum (FBS) and 1% penicillin–streptomycin, while THLE-2 cells were maintained in the BEGM Bullet Kit medium (CC-3170, Lonza Bioscience, Walkersville, MD, USA), which contains 500 mL of basal medium and separately packaged frozen supplements. Human THP-1 monocytic cells were purchased from the Cell Bank of the Chinese Academy of Sciences (Shanghai, China) and cultured in RPMI 1640 medium with 10% FBS. The THP-1 cells were treated with 100 ng/mL phorbol 12-myristate 13-acetate (PMA, Sigma-Aldrich, MO, USA) for 24 h to generate adherent macrophages. All cells were cultured in a humidified incubator at 37 °C with 5% CO_2_. Cells were passaged when they reached 80–90% confluence. For experiments, cells were seeded at 8 × 10^4^ cells/mL into 96-, 24-, 12-, or 6-well plates and cultured for 20 h, then synchronized in 2% FBS medium for 10 h. For treatment, the model group was exposed to 600 μM FFA, the drug group received 600 μM FFA together with 10 μM CuF, and the control group received an equivalent volume of 5% bovine serum albumin (BSA). Each condition was set up in 3–5 replicate wells and incubated for 36 h at 37 °C in 5% CO_2_.

### 2.2. Plant Material and Preparation of Cuspidatyl Ferulate (CuF)

CuF was isolated from the aerial parts of *H. cuspidatus* Borris, as described in our previous publication [15]. The isolated compound was stored in an air-tight and light-protected container until use. The purity was determined by HPLC (≥98%). For experiments, 10 mg of CuF was dissolved in 500 μL of dimethyl sulfoxide (DMSO) to make a 20 mg/mL stock solution. The CuF stock was pre-diluted in medium before each treatment, and the final DMSO concentration was kept below 0.1%. 

### 2.3. Free Fatty Acids (FFAs) Solution Preparation

The FFA solution was prepared by mixing oleic acid (O1008, Sigma-Aldrich, St. Louis, MO, USA) and palmitic acid (P5585, Sigma-Aldrich, St. Louis, MO, USA) at a molar ratio of 2:1, following previously described methods [16].

### 2.4. RNA Isolation and Quantitative Reverse Transcription PCR Experiments

Total RNA was extracted using TRIzol reagent (Invitrogen, Carlsbad, CA, USA), and cDNA was synthesized with the TransScript SuperMix kit (TransGen Biotech, Beijing, China). Quantitative PCR (qPCR) was performed using SYBR Green Master Mix (Roche, Basel, Switzerland). Relative expression levels were calculated using the comparative 2^−ΔΔCt^ method with 18sRNA as the internal control. The human gene symbols, full names, and primer sequences used in this study are summarized in Table 1.

### 2.5. Western Blot Analysis

Nuclear, cytoplasmic, and total protein extraction and Western blotting were performed as previously described [17], with minor modifications. Total proteins were extracted using RIPA buffer, and nuclear/cytoplasmic proteins were isolated using a commercial extraction kit (Vazyme, Nanjing, China) according to the manufacturers’ instructions. Protein concentrations were determined using a BCA assay (Beyotime, Shanghai, China).

Equal amounts of protein (15 µg) were separated by SDS–PAGE (8–15% gels) and transferred onto polyvinylidene fluoride (PVDF) membranes (GE Healthcare Life Science, Marlborough, MA, USA). Membranes were blocked with 5% non-fat milk in TBST for 1 h at room temperature, followed by overnight incubation at 4 °C with the following primary antibodies (all from Proteintech, Rosemont, IL, USA): rabbit anti-Nrf2 (1:1000) (catalog number 16396-1-AP), rabbit anti-Keap1 (1:1000) (catalog number 10503-2-AP), rabbit anti-Histone H3 (1:2000) (catalog number 17168-1-AP), and mouse anti-GAPDH (1:1000) (catalog number 60004-1-Ig).

After washing, membranes were incubated with HRP-conjugated goat anti-mouse IgG (H+L) (catalog number SA00001-1) or goat anti-rabbit IgG (H+L) (catalog number SA00001-2) secondary antibodies (1:5000; Proteintech, Rosemont, IL, USA) for 1 h at room temperature. Protein bands were visualized using an enhanced chemiluminescence (ECL) system (Thermo Fisher Scientific, Waltham, MA, USA) and quantified with ImageJ software, version 1.54. Histone H3 and GAPDH were used as nuclear and cytoplasmic loading controls, respectively.

### 2.6. Co-Immunoprecipitation (Co-IP) Assay

Co-IP was performed to evaluate the interaction between Keap1 and Nrf2 using Protein A–agarose (Sigma-Aldrich, St. Louis, MO, USA) according to the manufacturer’s protocol. Briefly, treated HepG2 cells were lysed on ice in lysis buffer and centrifuged at 10,000× *g* for 10 min at 4 °C. The supernatant was precleared with Protein A–agarose for 30 min at 4 °C to minimize nonspecific binding, followed by centrifugation at 1000× *g* for 5 min. The precleared lysate was then incubated with an anti-Nrf2 antibody or control rabbit IgG (Beyotime, Shanghai, China, catalog number A7001) for 1 h at 4 °C, followed by the addition of Protein A–agarose and gentle rotation overnight at 4 °C. The immunocomplexes were collected by centrifugation at 1000× *g* for 5 min at 4 °C, washed four times with lysis buffer, and resuspended in SDS loading buffer. Samples were boiled for 5 min, separated by 15% SDS–PAGE, and analyzed by Western blotting with the indicated antibodies [18].

### 2.7. Immunofluorescence (IF) Assay

IF staining was performed to evaluate the effect of CuF on Nrf2 nuclear translocation, as previously described [19]. Cells were fixed with 4% paraformaldehyde for 20 min and permeabilized with 0.2% Triton X-100 for 10 min. After blocking with 5% BSA for 30 min at room temperature, cells were incubated overnight at 4 °C with an anti–Nrf2 primary antibody (1:200). Following three washes with PBS, cells were incubated with secondary antibodies at a 1:500 dilution of Alexa488-conjugated anti-rabbit IgG (Proteintech Group, Rosemont, IL, USA) plus 1 μg/mL of DAPI (Sigma-Aldrich, Saint Louis, MO, USA) (for nuclear staining) for 5 min, in the dark at RT. Cells were washed four times with PBS (5 min each). Finally, fluorescence images were acquired using a BD Pathway™ Bioimager 855 high-content imaging system (BD Biosciences, San Jose, CA, USA) at 200× magnification.

### 2.8. Cell Viability Assay

Cell viability was evaluated using the [3-(4,5-dimetlthiazol-2-yl)-2,5-diphenyltetrazolium bromide (MTT) (Sigma-Aldrich, St. Louis, MO, USA)] MTT assay. HepG2 cells or THLE-2 cells were seeded in 96-well plates at a density of 8 × 10^3^ cells/well and cultured for 20 h, followed by serum starvation in 2% FBS medium for 10 h. Cells were then treated with various concentrations of CuF (1, 5, 10, and 20 μM) or co-treated with 600 μM FFA and 10 μM CuF for 36 h. After treatment, 20 μL of MTT solution (5 mg/mL) was added to each well and incubated for 4 h at 37 °C. The supernatant was removed, and 100 μL of DMSO was added to dissolve the formazan crystals. Plates were shaken for 10 min, and absorbance was measured at 490 nm using a microplate reader (Molecular Devices, San Jose, CA, USA). Cell viability was calculated as a percentage relative to the untreated control.

### 2.9. Intracellular ROS Measurement

Intracellular ROS levels were detected using a DCFH-DA fluorescent probe (Beyotime, Shanghai, China) according to the manufacturer’s instructions. After treatment, HepG2 cells were collected, washed with PBS, and incubated with DCFH-DA at 37 °C for 30 min in the dark. Fluorescence intensity was measured using a microplate reader (excitation/emission: 488/525 nm) or analyzed by flow cytometry (FlowJo LLC, Ashland, OR, USA, version 10). ROS levels were expressed as a percentage relative to the control. Fluorescence images were captured using an inverted fluorescence microscope.

### 2.10. Antioxidant Enzyme and Lipid Peroxidation Assays

Treated HepG2 cells were lysed on ice by sonication (25% amplitude, 7 s pulses, 6 s intervals, 10 cycles) in RIPA buffer (Beyotime, Shanghai, China) supplemented with protease and phosphatase inhibitor cocktail, followed by centrifugation at 12,000× *g* for 10 min at 4 °C. Protein concentration was determined using a BCA assay kit (Beyotime, Shanghai, China).

SOD Activity: Measured using the WST-8 method. Briefly, 20 μL of the supernatant was mixed with the working solution, incubated at 37 °C for 30 min, and the absorbance was recorded at 450 nm.

CAT Activity: Determined using the ammonium molybdate method. A 10 μL aliquot of the supernatant was mixed with 10 μL of H_2_O_2_ and 30 μL of buffer, incubated at 25 °C for 5 min, and the absorbance was measured at 405 nm.

GSH-Px Activity: Assessed according to the manufacturer’s instructions, and absorbance was measured at 412 nm.

MDA Content: Determined by the thiobarbituric acid metho. A 100 μL aliquot of the supernatant was mixed with the working solution, boiled for 15 min, cooled to room temperature, centrifuged, and the absorbance was measured at 450 nm.

All enzyme activities and MDA contents were normalized to total protein levels and expressed as U/mg protein or μmol/mg protein relative to the control group.

### 2.11. Oil Red O Staining

HepG2 cells were seeded on 24-well plates containing sterile coverslips at a density of 1 × 10^4^ cells/well and treated for 36 h. After treatment, cells were washed twice with PBS, fixed in calcium-formaldehyde for 10 min, and rinsed with distilled water. Cells were then pretreated with 60% isopropanol for 2 s and stained with 200 μL Oil Red O working solution for 25 min at room temperature. After brief differentiation with 60% isopropanol for 5 s and rinsing with distilled water, nuclei were counterstained with Mayer’s hematoxylin for 0.5–1 min. Coverslips were mounted with glycerol, and images were captured using a light microscope.

### 2.12. Lipid Analysis

Cellular total TC and TG levels were quantified using commercial assay kits (Nanjing Jiancheng Bioengineering Institute, Nanjing, China) following the manufacturer’s instructions. Absorbance was measured with a microplate reader, and lipid concentrations were calculated from standard curves and normalized to total protein content.

### 2.13. Molecular Docking and Molecular Dynamics Simulation (MDS)

The X-ray crystal structure of human Keap1 (amino acids 321–609, PDB ID: 4XMB) [20] was obtained from the RCSB Protein Data Bank [21]. Molecular docking between CuF and Keap1 was performed using AutoDock 1.5.6 [22]. Prior to docking, the receptor was processed in PyMOL [23] to remove solvent and water molecule. Hydrogen atoms and Gasteiger charges were added using AutoDock tools, and ligand chirality was corrected. The prepared receptor and ligand were saved in PDBQT format.

A docking grid box was defined to encompass the Keap1 ligand-binding pocket, centered on the known active site. Default parameters were applied except for the grid dimensions, which were adjusted to fully cover the active site. Docking results were ranked according to binding energy scores [24], and the lowest-energy conformation was selected for subsequent MDS.

MDS were carried out using Amber20 [25]. The GAFF force field [26] was used for CuF, and the ff14SB force field [27] was used for Keap1. Protonation states were assigned using the tleap module, which also added hydrogen atoms. The 3D structure of CuF was optimized, and its electrostatic potential (ESP) was computed using Gaussian 09 at the B3LYP/6-31G(d) level of theory [28]. Atomic partial charges were obtained using the RESP method implemented in Antechamber. The solvated Keap1–CuF complex was equilibrated and simulated under an NPT ensemble (constant pressure and temperature) for 100 ns. Trajectories were recorded every 1 ps for analysis.

To further validate docking results, GOLD 5.2 [29] was used to evaluate receptor–ligand binding affinity and calculate GoldScore values [30]. Before docking, all water molecules and co-crystallized ligands were removed from the Keap1 structure, and missing hydrogens were added. The binding site was defined as a 6 Å radius around the co-crystallized ligand. Thirty genetic algorithm runs were performed with early termination disabled. Docking poses were ranked by GoldScore, with higher scores indicating stronger predicted affinity. All docking interactions were visualized using PyMOL 2.5.

### 2.14. Expression, Purification and Validation of Recombinant Human Keap1 and Keap1-Mut Proteins

Codon-optimized genes encoding recombinant human Keap1 (amino acids 321–609) and the corresponding mutant Keap1-mut (R415A and S363A, Table S1) were synthesized by GeneWiz Biotech (Suzhou, China) and cloned into pCOLD1 vector via NdeI/BamHI sites, generating N-terminal 6×His-tagged constructs for expression in *E. coli* BL21(DE3) [31,32]. The complete coding sequences are provided in Supplementary Materials, Section S1, with mutated residues clearly highlighted. Transformed cells were cultured, and expression was induced with isopropyl β-D-1-thiogalactopyranoside (IPTG) under optimized conditions (temperature: 15–30 °C; IPTG: 0.05–1 mM; induction time: 10–24 h). The highest soluble yield was obtained at 20 °C with 0.2 mM IPTG for 22 h, producing ~37 kDa proteins. Soluble fractions were used for downstream purification.

Cells were harvested by centrifugation, resuspended in lysis buffer (50 mM Tris-HCl, pH 8.0, 300 mM NaCl, 10 mM imidazole), and lysed by sonication. The lysate was clarified by centrifugation, and soluble proteins were purified using a His-tag affinity chromatography kit (Beyotime, Shanghai, China). Bound proteins were eluted using an imidazole gradient. Purity was confirmed by SDS–PAGE and Coomassie Brilliant Blue staining, and identity was verified by Western blot with anti-His (Proteintech, Rosemont, IL, USA, catalog number 66005-1-Ig) and anti-Keap1 antibodies. The purified proteins were desalted, concentrated by ultrafiltration, and lyophilized, yielding ~15 mg of protein per 0.5 L culture at ~0.5 mg/mL.

### 2.15. Capillary Electrophoresis (CE) Analysis

Protein purity and homogeneity were assessed by CE as described previously [33,34]. Protein samples were diluted to 0.1 mg/mL in 50 mM phosphate buffer (pH 7.4) containing 10% (*v/v*) ethylene glycol to minimize adsorption. CE was performed using a fused-silica capillary (50 μm i.d., 50 cm total length, 40 cm effective length) with a 50 mM phosphate running buffer (pH 7.4). An applied voltage of 25 kV was used, and detection was performed at 214 nm. Samples were injected electrokinetically for 5 s at 10 kV. Data were collected and processed using ChemStation software (Agilent Technologies, Santa Clara, CA, USA; version Rev. B.04.03) [35].

### 2.16. Statistical Analysis

Statistical analyses were performed using GraphPad Prism 10.1.2 (GraphPad Software, San Diego, CA, USA). All data were presented as mean ± SD. Two-tailed Student’s *t*-tests were used to compare data between two groups maintaining normal distributions. One-way ANOVA was used to compare multiple groups. A *p*-value less than 0.05 was considered statistically significant.

## 3. Results

### 3.1. CuF Mitigates FFA-Induced Viability Loss in HepG2 and THLE-2 Cells

**Figure 1:**
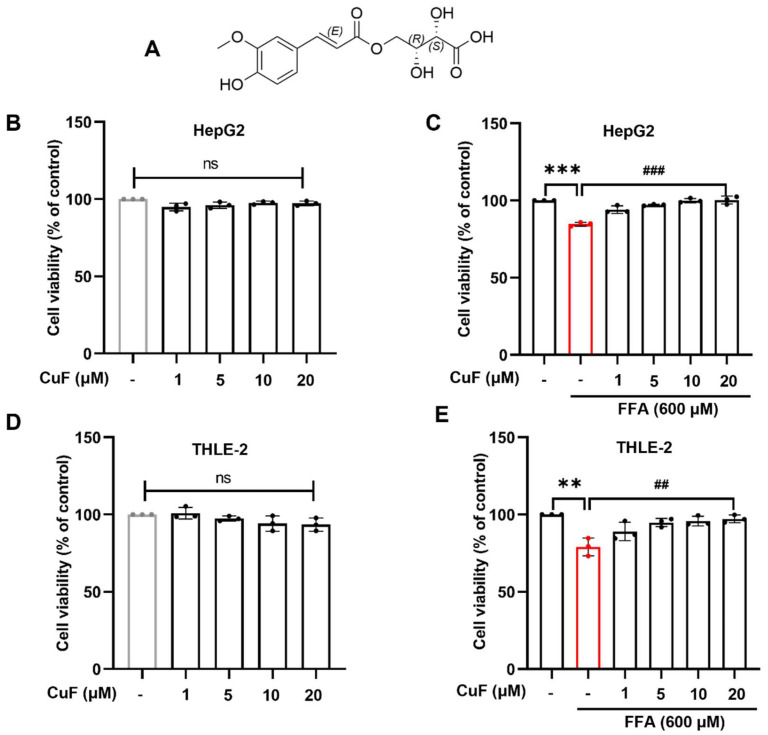
CuF protects hepatocytes from FFA-induced lipotoxicity in a dose-dependent manner. (**A**) Chemical structure of CuF. (**B**) CuF (0–20 μM) showed no cytotoxicity in HepG2 cells after 36 h treatment. (**C**) Co-treatment with CuF alleviated 600 μM FFA-induced viability loss in HepG2 cells in a dose-dependent manner. (**D**,**E**) Similar protective effects of CuF were observed in normal THLE-2 hepatocytes. Color coding: In panels (**B**,**D**), gray bars denote the control group, and black bars denote cells treated with CuF alone. In panels (**C**,**E**), black bars denote the control group or the FFA + CuF co-treatment group, and red bars denote the FFA-treated group. Data are presented as mean ± SD from three independent experiments. ns, not significant; ** *p* ≤ 0.01, *** *p* ≤ 0.001 versus control group; ^##^ *p* ≤ 0.01, ^###^ *p* ≤ 0.001 versus FFA group. FFA group.

To investigate the protective effect of CuF against FFA-induced lipotoxicity, we first established a model of oxidative injury in HepG2 hepatocytes using FFA (Figure S1A). Initial compound screening showed that CuF markedly enhanced SOD activity in FFA-treated HepG2 cells (Figure S1B), comparable to the positive control resveratrol (Figure S1B,C), suggesting that CuF exerts antioxidant activity. Based on this result, CuF (Figure 1A) was selected for further mechanistic studies.

The cytotoxicity of CuF alone was then evaluated by MTT assay. Treatment with CuF (0–20 μM) for 36 h did not affect HepG2 cell viability, which remained close to 100% (Figure 1B), indicating that CuF (up to 20 μM) is non-toxic and does not promote proliferation under basal conditions. In contrast, exposure to 600 μM FFA reduced HepG2 viability by approximately 15% (Figure S1B), confirming the successful establishment of oxidative stress–induced hepatocyte injury. Co-treatment with CuF significantly alleviated FFA-induced cytotoxicity in a dose-dependent manner. Compared with the FFA group, CuF treatment increased cell viability by 8.45%, 11.42%, 12.30%, and 14.33% at 1, 5, 10, and 20 μM, respectively (Figure 1C). Notably, 10 μM CuF restored cell viability to nearly control levels, indicating effective protection without stimulating cell growth.

To verify that this protective effect was not limited to the hepatoma-derived HepG2 model, we repeated the experiments in THLE-2 cells, an immortalized but non-tumorigenic human hepatocyte line. Consistently, CuF also alleviated FFA-induced viability loss in THLE-2 cells in a similar concentration-dependent manner (Figure 1D,E). These results demonstrate that CuF effectively mitigates FFA-induced oxidative damage in both hepatoma and non-tumorigenic hepatocyte models. Therefore, 10 μM CuF was selected for subsequent mechanistic investigations.

### 3.2. CuF Attenuates FFA-Induced ROS Generation in HepG2 Cells

**Figure 2:**
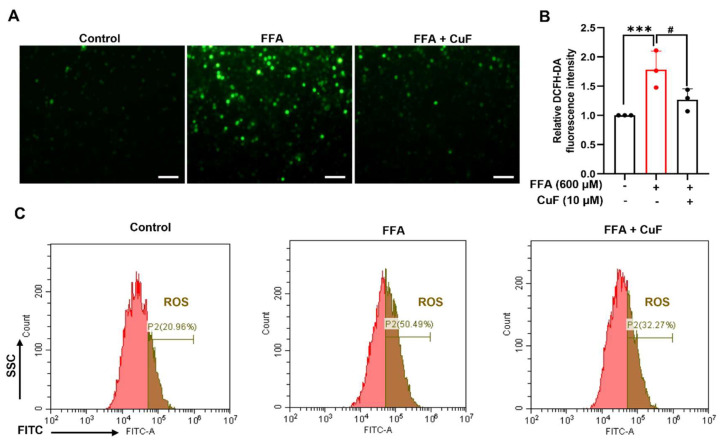
CuF attenuates FFA-induced ROS accumulation in HepG2 cells. (**A**) Representative fluorescence images of intracellular ROS detected using the DCFH-DA probe. Scale bar = 50 μm. (**B**) Quantification of DCF fluorescence intensity in different treatment groups. Fluorescence intensity was normalized to cell viability. (**C**) Flow cytometry analysis showing intracellular ROS levels in HepG2 cells under different treatments. Color coding: In panel (**B**), black bars denote the control group or the FFA + CuF co-treatment group, and red bars denote the FFA-treated group. Data are presented as mean ± SD (n = 3). *** *p* ≤ 0.001 versus control group; ^#^ *p* ≤ 0.05, versus FFA-treated group.

To further evaluate the antioxidant effect of CuF, we examined its ability to counteract FFA-induced intracellular ROS accumulation in HepG2 cells. Cells were loaded with the DCFH-DA fluorescent probe to visualize and quantify ROS levels. Exposure to 600 μM FFA caused a pronounced increase in ROS fluorescence compared with the untreated control, confirming the establishment of oxidative stress (Figure 2A,B). Co-treatment with 10 μM CuF markedly reduced ROS fluorescence intensity, restoring it close to baseline levels. Flow cytometry analysis further validated these observations, showing a significant decrease in the proportion of ROS-positive cells following CuF treatment compared with FFA alone (Figure 2C). Collectively, these results demonstrate that CuF effectively suppresses FFA-induced ROS generation and mitigates oxidative stress in hepatocytes.

### 3.3. CuF Restores Antioxidant Defense in HepG2 Cells Under FFA-Induced Oxidative Stress

**Figure 3:**
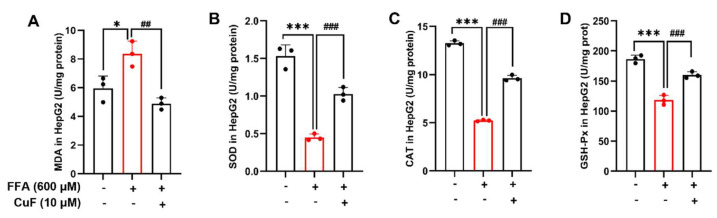
CuF restores antioxidant defense capacity in FFA-treated HepG2 cells. (**A**) MDA content in HepG2 cells treated with FFA and/or CuF. (**B**–**D**) Activities of antioxidant enzymes SOD, CAT, and GSH-Px in HepG2 cells treated with FFA and/or CuF. Color coding: Black bars denote the control group or the FFA + CuF co-treatment group, and red bars denote the FFA-treated group. Data are presented as mean ± SD (n = 3). * *p* ≤ 0.05, *** *p* ≤ 0.001 versus control group; ^##^ *p* ≤ 0.01, ^###^ *p* ≤ 0.001 versus FFA-treated group.

To investigate whether CuF enhances endogenous antioxidant defenses, we measured lipid peroxidation and the activities of major antioxidant enzymes, including SOD, CAT, and GSH-Px [36]. Exposure to 600 μM FFA significantly elevated MDA levels in HepG2 cells, indicating increased lipid peroxidation (Figure 3A). Co-treatment with 10 μM CuF markedly decreased MDA content, suggesting effective suppression of oxidative membrane damage. In parallel, FFA treatment significantly inhibited SOD, CAT, and GSH-Px activities, whereas CuF co-treatment restored these enzyme activities to levels comparable to those in control cells (Figure 3B–D). Together, these findings indicate that CuF alleviates FFA-induced oxidative stress by reinforcing the intracellular antioxidant defense system and preventing lipid peroxidation.

### 3.4. CuF Protects HepG2 Cells from FFA-Induced Lipotoxicity by Rebalancing Fatty Acid Metabolism

**Figure 4:**
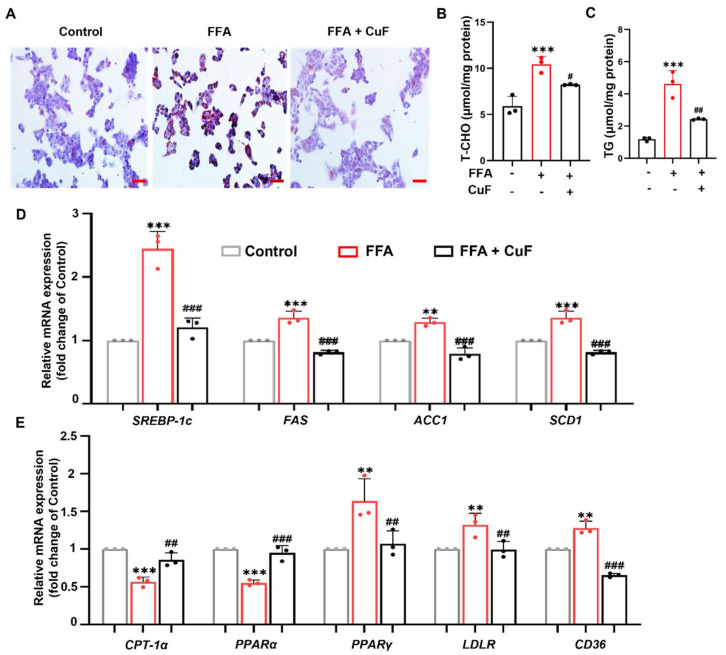
CuF alleviates FFA-induced lipid accumulation and rebalances fatty acid metabolism in HepG2 cells. (**A**) Representative Oil Red O staining showing intracellular lipid droplets in different treatment groups. Scale bar = 50 μm. (**B**,**C**) Quantification of intracellular TC and TG levels. (**D**) Relative mRNA expression of lipogenic genes (*SREBP-1c*, *FAS*, *ACC1*, and *SCD1*) in HepG2 cells. (**E**) Relative mRNA expression of fatty acid oxidation–related genes (*CPT-1α*, *PPARα*) and lipid uptake–related genes (*PPARγ*, *LDLR*, and *CD36/FAT*). Color coding: In panels (**B**,**C**), black bars denote the control group or the FFA + CuF co-treatment group, and red bars denote the FFA-treated group. In panels (**D**,**E**), gray bars denote the control group, red bars denote the FFA-treated group, and black bars denote the FFA + CuF co-treatment group. Data are presented as mean ± SD (n = 3). ** *p* ≤ 0.01, *** *p* ≤ 0.001 versus control group; ^#^ *p* < 0.05, ^##^ *p* ≤ 0.01, ^###^ *p* ≤ 0.001 versus FFA-treated group.

To evaluate whether CuF alleviates FFA-induced lipid metabolic stress, intracellular lipid accumulation was first assessed by Oil Red O staining. Exposure to 600 μM FFA markedly increased both the number and size of lipid droplets, reflecting excessive lipid deposition and steatosis, consistent with previous reports [37]. Co-treatment with 10 μM CuF visibly reduced lipid droplet accumulation, indicating an effective suppression of FFA-induced lipid overload (Figure 4A). Quantitative biochemical analysis further confirmed these findings. FFA exposure significantly elevated intracellular TC and TG levels, whereas CuF co-treatment reduced both TC and TG concentrations toward baseline values (Figure 4B,C).

To explore the molecular mechanisms underlying these protective effects, we next examined the effect of CuF on fatty acid metabolism–related gene expression. Previous studies have shown that certain phenolic compounds inhibit the nuclear translocation of sterol regulatory element-binding protein 1c (SREBP-1c) through activation of AMP-activated protein kinase (AMPK) at Ser372, thereby reducing hepatic lipogenesis and improving steatosis [38]. Inspired by these findings, we investigated whether CuF modulates a similar pathway under FFA stress. CuF treatment significantly downregulated *SREBP-1c* mRNA expression and its downstream lipogenic targets, including fatty acid synthase (*FAS*), acetyl-CoA carboxylase 1 (*ACC1*), and stearoyl-CoA desaturase 1 (*SCD1*) (Figure 4D). Concurrently, CuF enhanced the expression of genes involved in fatty acid β-oxidation, such as carnitine palmitoyltransferase 1α (*CPT-1α*) and peroxisome proliferator-activated receptor α (*PPARα*), suggesting a shift toward enhanced oxidative metabolism. Moreover, CuF co-treatment increased the expression of genes associated with lipid transport and uptake, including peroxisome proliferator-activated receptor γ (*PPARγ*), low-density lipoprotein receptor (*LDLR*), and fatty acid translocase *CD36/FAT* (Figure 4E), implying improved lipid turnover and reduced intracellular lipotoxicity.

Taken together, these results demonstrate that CuF protects HepG2 cells from FFA-induced steatosis by rebalancing lipid metabolism. CuF reduces lipid synthesis while promoting fatty acid oxidation and uptake, thereby restoring metabolic homeostasis and mitigating oxidative stress.

### 3.5. CuF Activates the Nrf2–Keap1 Pathway to Mitigate FFA-Induced Oxidative Stress in HepG2 Cells

**Figure 5:**
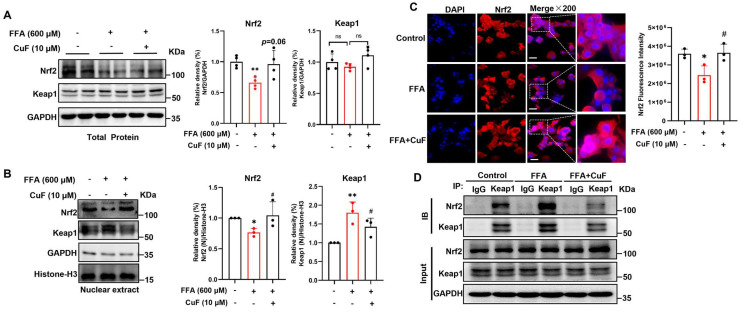
CuF activates the Nrf2–Keap1 pathway in FFA-treated HepG2 cells. (**A**) Western blot analysis of total Nrf2 and Keap1 protein levels in whole-cell lysates following the indicated treatments. (**B**) Western blot analysis of nuclear Nrf2 and Keap1 protein levels. (**C**) IF staining of Nrf2 (red) and nuclei (DAPI, blue) in HepG2 cells, scale bar = 50 μm. (**D**) Co-IP analysis of the interaction between Nrf2 and Keap1. Color coding: In panels (**A**–**C**), black bars denote the control group or the FFA + CuF co-treatment group, and red bars denote the FFA-treated group. Data are representatives of three independent experiments and are shown as mean ± SD. * *p* ≤ 0.05, ** *p* < 0.01 versus control group; ^#^ *p* ≤ 0.05 versus FFA-treated group.

The findings described above indicate that CuF alleviates FFA-induced lipotoxicity by suppressing SREBP-1c–mediated fatty acid biosynthesis, enhancing PPAR*γ*-dependent lipid uptake, and promoting CPT-1*α*–mediated fatty acid oxidation. In parallel, CuF increased the activity of key antioxidant enzymes. These observations suggested potential involvement of the Nrf2–Keap1 signaling pathway, a central regulator of cellular redox homeostasis [39]. Consistently, transcriptomic profiling revealed that CuF co-treatment restored the expression of genes related to mitochondrial oxidative phosphorylation and antioxidant defense, pointing toward activation of intrinsic redox-protective networks. Given that Nrf2 orchestrates cellular antioxidant responses and maintains mitochondrial homeostasis [16,40,41], we next investigated whether CuF modulates the Nrf2–Keap1 signaling axis. Western blot analysis of whole-cell lysates showed that CuF co-treatment partially counteracted the FFA-induced reduction in total Nrf2 protein levels. Although an upward trend was observed, the increase did not reach statistical significance under the current experimental conditions (n = 4, *p* = 0.06). In contrast, total Keap1 protein expression remained unchanged across treatment groups (Figure 5A). To further characterize CuF-mediated regulation of the Keap1–Nrf2 axis, nuclear fractions were examined. Analysis of nuclear extracts revealed that CuF significantly enhanced nuclear Nrf2 accumulation compared with FFA alone, indicating promotion of Nrf2 nuclear translocation. while simultaneously reducing nuclear Keap1 levels, consistent with disruption of the Keap1–Nrf2 complex (Figure 5B).

IF analysis supported the regulatory effects of CuF on overall Nrf2 abundance in HepG2 cells (Figure 5C). FFA markedly reduced Nrf2 fluorescence intensity, whereas CuF co-treatment restored the signal. Due to the resolution limitations of the BD Pathway™ system, these images primarily reflect total cellular Nrf2 levels rather than clearly distinguishing nuclear versus cytoplasmic localization. Although nuclear Nrf2 staining can be observed in some magnified regions, the Western blot analyses provide more definitive evidence of Nrf2 nuclear accumulation following CuF treatment (Figure 5B). The quantitative fluorescence analysis remains consistent with these biochemical data, showing FFA-induced suppression of Nrf2 and its recovery with CuF. To directly assess whether CuF affects Keap1–Nrf2 binding, Co-IP was performed. CuF treatment substantially decreased the amount of Keap1 bound to Nrf2 relative to the FFA group (Figure 5D), suggesting that CuF interferes with the inhibitory Keap1–Nrf2 interaction. By destabilizing the Keap1–Nrf2 complex rather than altering total protein levels, CuF facilitates Nrf2 release from Keap1-mediated ubiquitination and degradation. This allows Nrf2 to accumulate in the nucleus and activate transcription of downstream antioxidant genes, including SOD, CAT, and GSH-Px. Collectively, these results demonstrate that CuF activates the Nrf2–Keap1 signaling pathway by disrupting Keap1–Nrf2 binding, thereby promoting Nrf2 nuclear translocation and strengthening the cellular antioxidant defense system against FFA-induced oxidative stress.

### 3.6. CuF Specifically Binds Keap1 and Disrupts the Nrf2–Keap1 Interaction

**Figure 6:**
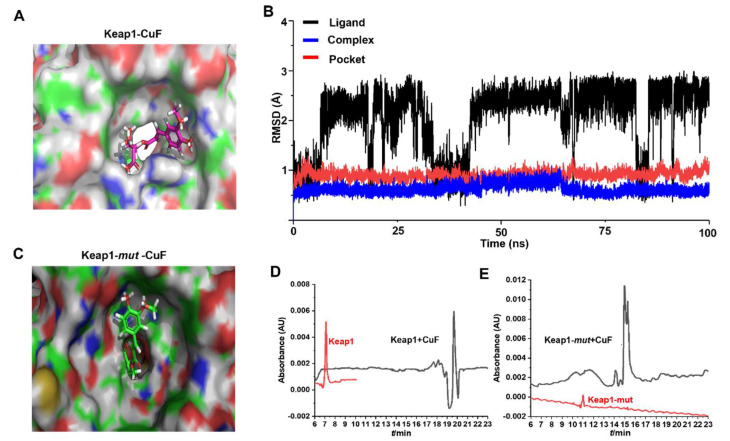
CuF specifically binds to the Kelch domain of Keap1 and disrupts the Keap1–Nrf2 interaction. (**A**) Molecular docking model showing the binding of CuF within the Kelch domain of Keap1. (**B**) Root mean square deviation (RMSD) trajectories of the CuF–Keap1 complex during a 100 ns molecular dynamics simulation, demonstrating structural stability. (**C**) Binding mode of CuF with mutant Keap1 (Keap1-mut; Arg415 and Ser363 substituted with alanine, R415A/S363A, residues 321–609). (**D**,**E**) Capillary electrophoresis (CE) analysis of CuF binding to wild-type Keap1 (**D**) and Keap1-mut (**E**).

To investigate the molecular mechanism by which CuF activates the Nrf2–Keap1 pathway, we first examined the interaction between CuF and Keap1 using molecular docking and molecular dynamics simulations (MDS). Docking results revealed favorable binding of CuF within the Kelch domain of Keap1 (Figure 6A), with a GOLD score of 66.04 and an ASP score of 26.07 (Table 2), indicating strong ligand–receptor interactions.

To assess complex stability, a 100 ns MDS was performed using Amber20. RMSD trajectories (Figure 6B) stabilized within the first 2 ns, with minor fluctuations mainly attributed to flexible regions of CuF. RMSF analysis (Figure S2) indicated that residues within the active pocket (e.g., 363–366, 380, 414–418, 461–465, 475, 508–512, 555–559, 602) exhibited minimal conformational flexibility, supporting stable ligand binding. MM/PBSA binding free energy calculations (Figure S3) suggested that van der Waals interactions were the primary driving force, complemented by electrostatic interactions and solvation energies. Key residues contributing to CuF binding included Arg415, Gly509, Tyr525, Gly462, Gly603, Ile461, Ser508, Gly364, Arg483, Ser363, and Phe478 (Table S2), consistent with docking predictions. Notably, CuF occupied the Nrf2-binding region of Keap1, suggesting potential disruption of the Keap1–Nrf2 complex.

To validate the predicted CuF binding site, Arg415 and Ser363 of Keap1 (amino acids 321–609) were substituted with alanine to generate Keap1-mut (R415A/S363A, Table S1). Docking of CuF to Keap1-mut revealed a markedly altered binding mode (Figure 6C), with GOLD and ASP scores decreasing to 42.97 and 11.23, respectively (Table 2), indicating that these residues are critical for CuF binding.

Experimental validation was then performed using CE to measure changes in protein migration upon CuF binding. Recombinant Keap1 (aa 321–609) and Keap1-mut (R415A/S363A) were expressed, purified, and used in these assays (Figure S4). For wild-type Keap1, the migration time shifted from 7.15 min to 19.67 min after CuF addition, demonstrating strong binding (Figure 6D). In contrast, Keap1-mut exhibited a smaller shift from 10.99 min to 14.97 min (Figure 6E), reflecting weakened interaction due to mutation of key residues. These results confirm that CuF specifically binds critical residues in Keap1, consistent with molecular docking and dynamics predictions.

**Figure 7:**
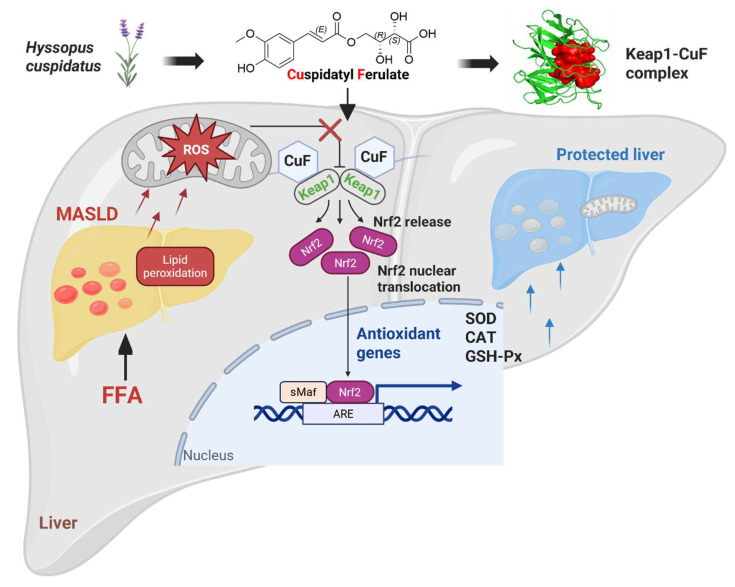
Proposed mechanism of the protective effect of CuF against FFA-induced oxidative stress. CuF directly binds to the Nrf2-binding site of Keap1, disrupting the Keap1–Nrf2 complex and promoting Nrf2 nuclear translocation. Activated Nrf2 enhances the transcription of antioxidant enzymes, thereby restoring cellular redox balance and protecting hepatocytes from FFA-induced oxidative damage.

Taken together, these findings indicate that CuF directly interacts with the Nrf2-binding region of Keap1, disrupting the Keap1–Nrf2 complex and facilitating Nrf2 nuclear translocation. This interaction likely underlies CuF’s ability to enhance antioxidant enzyme expression and mitigate FFA-induced oxidative stress (Figure 7).

## 4. Discussion

Identifying safe and effective therapeutic targets for MASLD remains a significant challenge. In this study, we investigated CuF, a newly identified natural phenolic acid derived from *H. cuspidatus* and demonstrated that it exerts strong antioxidant and anti-lipotoxic effects in an FFA-induced HepG2 cell model. CuF effectively reduced ROS accumulation, enhanced the activity of endogenous antioxidant enzymes, and attenuated intracellular lipid deposition. These findings highlight the therapeutic potential of CuF as a natural product-based candidate for MASLD intervention.

MASLD is a complex systemic disease involving insulin resistance, lipotoxicity, oxidative stress, inflammation, apoptosis, impaired autophagy, and mitochondrial dysfunction [42]. Although the thyroid hormone receptor-β agonist Resmetirom has recently been approved by the FDA for treatment of MASH, the overall lack of effective therapeutics underscores the continued need for novel approaches. Natural products have emerged as promising sources of hepatoprotective agents, owing to their multi-target metabolic, antioxidant, and anti-inflammatory activities, as well as their ability to modulate the gut–liver axis [43]. Compounds such as taxifolin [44], silymarin [45], curcumin [46], and resveratrol [47] have shown beneficial effects against hepatic steatosis in preclinical models. Nonetheless, the clinical efficacy of many polyphenols remains inconclusive, and their mechanisms of action are not fully defined [48].

Given the well-recognized antioxidant properties of polyphenols, we first evaluated the effects of CuF on oxidative stress. CuF significantly reduced ROS production in FFA-treated hepatocytes and increased the activities of SOD, CAT, and GSH-Px, indicating a robust enhancement of endogenous antioxidant defenses. In addition, CuF markedly decreased FFA-induced lipid accumulation. This reduction was associated with downregulation of lipogenic genes (*SREBP1c*, *FAS*, *ACC1*, and *SCD1*) and normalization of fatty acid oxidation and uptake-related genes (*CPT1A*, *PPARα*, *LDLR*, and *CD36*). Importantly, CuF alone did not affect cell viability or disrupt basal lipid metabolic pathways in HepG2 cells (Figure S5) suggesting that its regulatory actions become most apparent under conditions of metabolic stress. Notably, CuF exerted consistent protective effects across multiple cell systems cultured in distinct media formulations. Similar cytoprotective and antioxidant responses were observed in HepG2 cells cultured in DMEM, THLE-2 cells maintained in BEGM, and PMA-stimulated THP-1 macrophages grown in RPMI (Figure S6). This cross-model consistency suggests that the biological activity of CuF is primarily compound-specific rather than medium-dependent, further supporting its potential applicability across different hepatic and immune cell contexts under lipotoxic stress.

To further elucidate the molecular basis of CuF’s antioxidant effects, we investigated whether CuF directly targets the Nrf2–Keap1 pathway. Molecular dynamics simulations revealed that CuF enters the Keap1 binding pocket, occupying its central cavity and interfering with the Keap1–Nrf2 interaction. This prediction aligns with previous studies showing that natural compounds such as ellagic acid, isoquercitrin, quercetin, thonningianin A, polypodisid, and rhodiogenin directly bind Keap1 and activate Nrf2 signaling [49,50,51]. Mutational analysis confirmed that CuF binds to residues Arg415 and Ser363, as mutation of these key sites significantly reduced CuF–Keap1 affinity. Together, these results support a model in which CuF disrupts Keap1-mediated Nrf2 sequestration, thereby promoting Nrf2 nuclear translocation and enhancing antioxidant gene expression.

Several limitations of our study should be acknowledged. First, the main experi-ments were conducted in HepG2 cells, an immortalized hepatoma line that does not fully recapitulate normal liver physiology. Although THLE-2 cells were included to partially address this concern, additional studies using primary hepatocytes or *in vivo* models will be essential to validate the therapeutic relevance of CuF. Second, the molecular simulations were performed using a partial Keap1 protein sequence, which may not fully capture all conformational states or accessory interaction surfaces; therefore, complementary biochemical validation using full-length Keap1 is warranted. Third, MASLD involves not only hepatocytes but also Kupffer cells, liver sinusoidal endothelial cells and hepatic stellate cells, which contribute substantially to inflammation and disease progression. Whether CuF impacts these non-parenchymal cell types remains to be determined. We preliminarily investigated the effect of CuF on macrophages using the THP-1 cell line. The MTT and flow cytometry results indicated that CuF alleviated the reduction in cell viability and attenuated ROS production induced by 36-hour FFA treatment in PMA-stimulated THP-1 cells (Figure S6), which is consistent with the findings in hepatocytes. However, the mechanism by which CuF mediates the antioxidant effect in macrophages, and whether it exerts similar functions in other non-parenchymal liver cells, require future investigation. Finally, although CuF was tested under non-physiological *in vitro* conditions, these findings establish a mechanistic basis for future pharmacological evaluation in preclinical models.

In summary, our study identifies CuF as a promising natural product with potent antioxidant and anti-lipotoxic properties. CuF reduces FFA-induced oxidative stress and lipid accumulation, in part by directly disrupting the Keap1–Nrf2 interaction. These findings provide a mechanistic foundation for further development of CuF as a potential therapeutic agent for MASLD. Future studies incorporating primary liver cell models, macrophages, and *in vivo* validation will be critical to further assess its efficacy, safety, and translational potential.

## 5. Conclusions

In conclusion, this study reveals that CuF, a novel phenolic acid, alleviated FFA-induced oxidative stress and lipid accumulation in HepG2 cells. By directly binds to Keap1, CuF disrupts the Keap1-Nrf2 protein interaction and activates the Nrf2-mediated antioxidant pathway. These results underscore the therapeutic potential of CuF for oxidative damage related diseases.

## Figures and Tables

**Table 1:** The primer sequences of qPCR in the study.

Gene Symbol	Full Name	Forward Primer (5′–3′)	Reverse Primer (5′–3′)
*ACC1*	Acetyl-CoA carboxylase 1	TTGATTCCTGGCTCTACCC	TCACTGCCTCTGAATACACA
*PPARG*	Peroxisome proliferator-activated receptor gamma	CTCATATCCGAGGGCCAAGG	TTGCCAAGTCGCTGTCATCT
*PPARA*	Peroxisome proliferator-activated receptor alpha	GCTTCGCAAACTTGGACCTG	ACCAGCATCCCGTCTTTGTT
*NRF2*	Nuclear factor erythroid 2–related factor 2	GAGCAAGTTTGGGAGGAGCT	TGGCTTCTGGACTTGGAACCC
*LDLR*	Low-density lipoprotein receptor	ATGACTGCCCAACTCCCATG	ACTGATGGGTGAAGTGCTGG
*SCD1*	Stearoyl-CoA desaturase 1	TTGATTCCTGGCTCTACCC	TCACTGCCTCTGAATACACA
*FAS*	Fatty acid synthase	CTTGGTCTTCTTTATTGGCAT	AGGAAAATTACAAATGGCCTT
*SREBP1*	Sterol regulatory element-binding protein 1	GCACTGAGGCAAAGCTGAAT	CCGACACCAGATCCTTCAGA
*CD36*	Cluster of differentiation 36	AACCTATTGGTCAAGCCAT	ATGTTTGCCTTCTCATCACC
*CPT1α*	Carnitine palmitoyltransferase 1 alpha	TGAGTGACTGGTGGGAGGAA	GCAGAGCAGAGGGGAATTGT
*18s RNA*	18S ribosomal RNA	GGAAGGGCACCACCAGGAGT	TGCAGCCCCGGACATCTAAG

**Table 2:** The molecular docking score of CuF with Keap1 and Keap1-mut.

Target	Gold Score Value	ASP Value
Keap1	66.04	26.07
Keap1-mut	42.97	11.23

Legend: Docking of CuF with wild-type Keap1 and mutant Keap1 was performed using GOLD software, version 5.2. The “Gold score value” represents the predicted binding affinity, and the “ASP value” is the internal scoring function output from GOLD. Higher values indicate more favorable binding.

## Data Availability

The original contributions presented in this study are included in the article and Supplementary Materials. Further inquiries can be directed to the corresponding authors.

## Supplementary Materials

The following supporting information can be downloaded at: https://www.mdpi.com/article/10.3390/antiox14121449/s1.

## Abbreviations

The following abbreviations are used in this manuscript:

ACC1	Acetyl-CoA carboxylase 1
ARE	Antioxidant Response Element
BSA	Bovine Serum Albumin
CAT	Catalase
CE	Capillary Electrophoresis
Co-IP	Co-immunoprecipitation
CPT1α	Carnitine palmitoyltransferase 1 alpha
CuF	Cuspidatyl ferulate (2,3-dihydroxy-4-carboxylic butyl (E)-4-[3-(4-hydroxy-3-methoxyphenyl)-2-propenoate])
DMEM	Dulbecco’s Modified Eagle Medium
ECL	Enhanced Chemiluminescence
FAS	Fatty acid synthase
FFA	Free Fatty Acid
FBS	Fetal Bovine Serum
GSH-Px	Glutathione Peroxidase
*H. cuspidatus*	*Hyssopus cuspidatus* Borris
IP	Immunofluorescence
IPTG	β-D-1-thiogalactopyranoside
Keap1	Kelch-like ECH-associated Protein 1
LDLR	Low-density lipoprotein receptor
MASH	Metabolic Dysfunction-Associated Steatohepatitis
MASLD	Metabolic Dysfunction-Associated Steatotic Liver Disease
MDS	Molecular Dynamics Simulation
MTT	[3-(4,5-dimethylthiazol-2-yl)-2,5-diphenyltetrazolium bromide]
NAFLD	Nonalcoholic Fatty Liver Disease
Nrf2/NRF2	Nuclear Factor Erythroid 2–Related Factor 2
PMA	Phorbol 12-myristate 13-acetate
PPARG	Peroxisome proliferator-activated receptor gamma
PPARA	Peroxisome proliferator-activated receptor alpha
ROS	Reactive Oxygen Species
SCD1	Stearoyl-CoA desaturase 1
SOD	Superoxide Dismutase
SREBP1	Sterol regulatory element-binding protein 1
TC	Total Cholesterol
TG	Triglyceride
CD36	Cluster of differentiation 36
18s RNA	18S ribosomal RNA
