# Supplementary material for: Cuspidatyl Ferulate, a Novel Phenolic Acid from Hyssopus cuspidatus Borris, Protects Hepatocytes Against Oxidative Damage via Keap1 Interaction
Source: Antioxidants (Basel). 2025 Dec 1;14(12):1449. doi: 10.3390/antiox14121449 (PMC12729638; doi:10.3390/antiox14121449)
Supplement: Supplementary file 1 [file antioxidants-14-01449-s001.zip › antioxidants-3979445-supplementary.pdf]

This file contains coding sequences and supplementary figures supporting the manuscript.

Section 1 – Protein Sequences

1. >Coding sequence of the Keap1 protein (Human, Wild-type, Kelch domain, aa 321–609) APKVGRLI YTAGGYFRQSLSYLEAYNPSDGTWLRLADLQVPRSGLAGCVVGGLLYAVGRNNSPDGNTDSSALD CYNPMTNQWSPCAPMSVPRNRIGVGVIDGHIYAVGGSHGCIHHNSVERYEPERDEWHLVAPMLTR RIGVGVAVLNRLYAVGGFDGTNRLNSAECYYPERNEWRMITAMNTIRSGAGVCVLHNCIYAAGGY DGQDQLNSVERDVETETWTFVAPMKHRRSALGITVHQGRIYVLGGYDGHTFLDSVECYDPDTDTW SEVTRMTSGRSGVGVAVT

2. >Coding sequence of the Keap1-*mut* protein (Human, Mutant Kelch domain, aa 321–609; R415A, S363A) APKVGRLIYTAGGYFRQSLSYLEAYNPSDGTWLRLADLQVPRAGLAGCVVGGLLYAVGGRNNSPD GNTDSSALDCYNPMTNQWSPCAPMSVPRNAIGVGVIDGHIYAVGGSHGCIHHNSVERYEPERDEW HLVAPMLTRRIGVGVAVLNRLLYAVGGFDGTNRLNSAECYYPERNEWRMITAMNTIRSGAGVCVL HNCIYAAGGYDGQDQLNSVERYDVETETWTFVAPMKHRRSALGITVHQGRIYVLGGYDGHTFLDS VECYDPDTDTWSEVTRMTSGRSGVGVAVT

Table S1. Keap1 point mutants used in this study

Protein	Original Residue	Position	Substitution
Keap1	Arg	415	Arg→Ala (R415A)
Keap1	Ser	363	Ser→Ala (S363A)

Legend: The table lists the Keap1 point mutants generated for functional studies. The “Original Residue” column indicates the wild-type amino acid at the given position, and “Substitution” shows the amino acid change introduced.

Section 2 – Supplementary Figures.

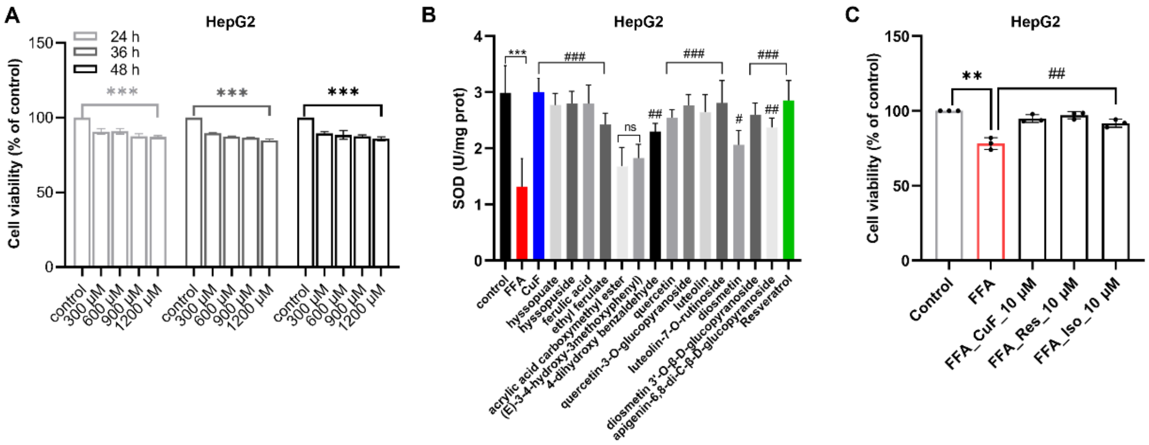

Figure S1. CuF protects hepatocytes from FFA-induced lipotoxicity in a dose-dependent manner. (A) Establishment of an FFA-induced oxidative stress model in HepG2 cells. Cells were treated with different FFA concentrations (300, 600, 900, 1200 μM) for 24, 36, or 48 h, and cell viability was measured to determine optimal stress conditions. (B) Screening of multiple monomeric compounds, including CuF, and Resveratrol (Res) for their ability to enhance SOD activity in HepG2 cells treated with 600 μM FFA for 24 h. (C) Comparative antioxidant effects of selected compounds (CuF, Res, and Isoacteoside (Iso)) in HepG2 cells under FFA treatment (600 μM, 24 h). Data are presented as mean ± SD from three independent experiments. ns, not significant; ** $p \le 0.01$, *** $p \le 0.001$ versus control group; # $p \le 0.05$, ## $p \le 0.01$, ### $p \le 0.001$ versus FFA group.

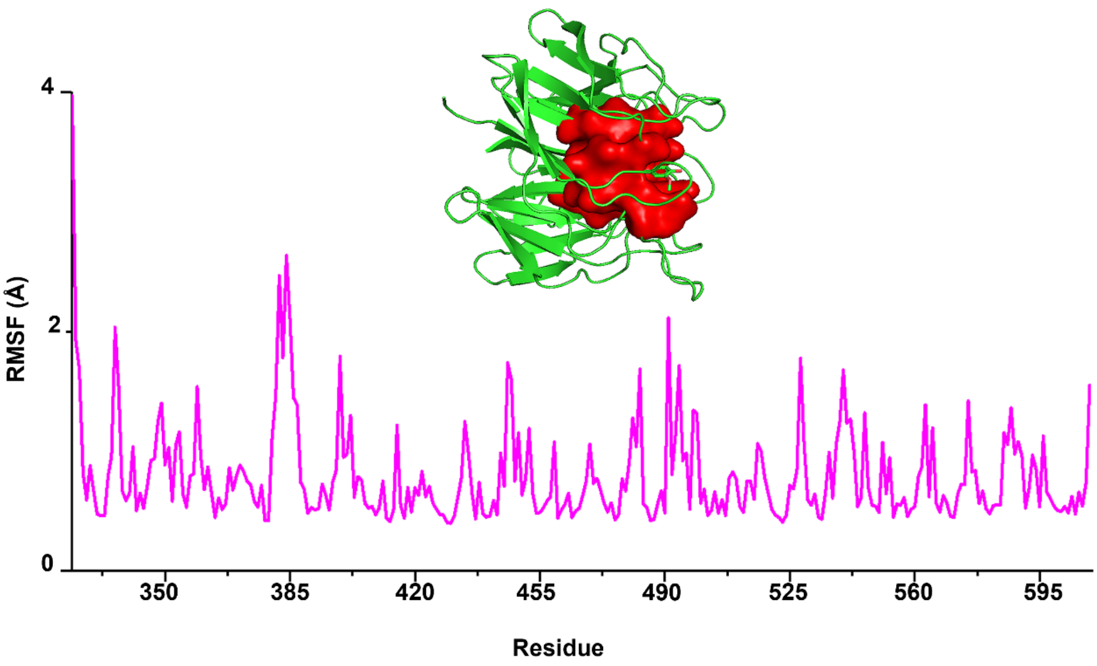

Figure S2. Root mean square fluctuation (RMSF) analysis of the CuF–Keap1 complex during 100 ns MDS. RMSF plots showing minimal flexibility within active-site residues (363–366, 380, 414–418, 461–465, 475, 508–512, 555–559, 602), confirming structural stability of the CuF–Keap1 complex.

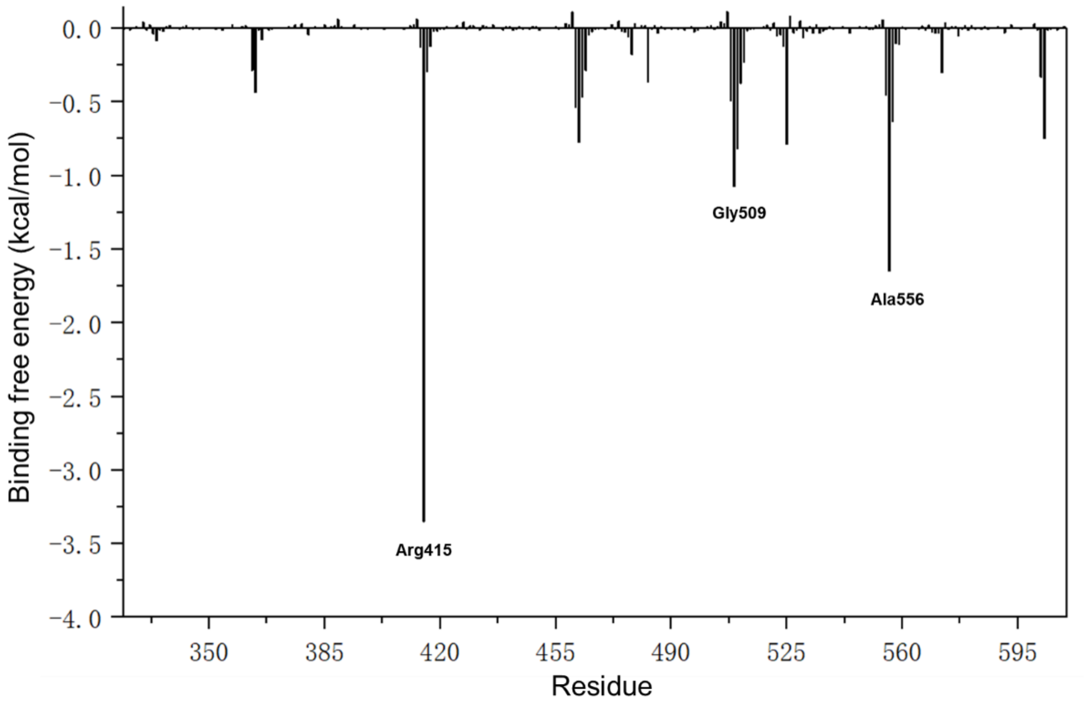

Figure S3. MM/PBSA binding free energy analysis of the CuF–Keap1 complex. Contribution of van der Waals, electrostatic, and solvation energies to the total binding free energy. van der Waals interactions were identified as the primary driving force for CuF–Keap1 complex stability.

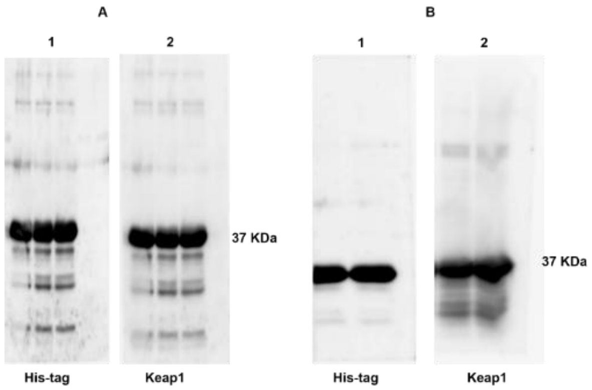

Figure S4. Western Blot analysis of recombinant protein Keap1 and Keap1-*mut*. A: Keap1. B: Keap1-*mut*. 1: Anti-His tag; 2: Anti-Keap1. The full length of Keap1 contains 624 amino acids; the recombinant protein used in this study corresponds to residues 321–609 and includes an N-terminal 6×His tag. The predicted molecular weight of this purified Keap1 fragment is approximately 32–33 kDa.

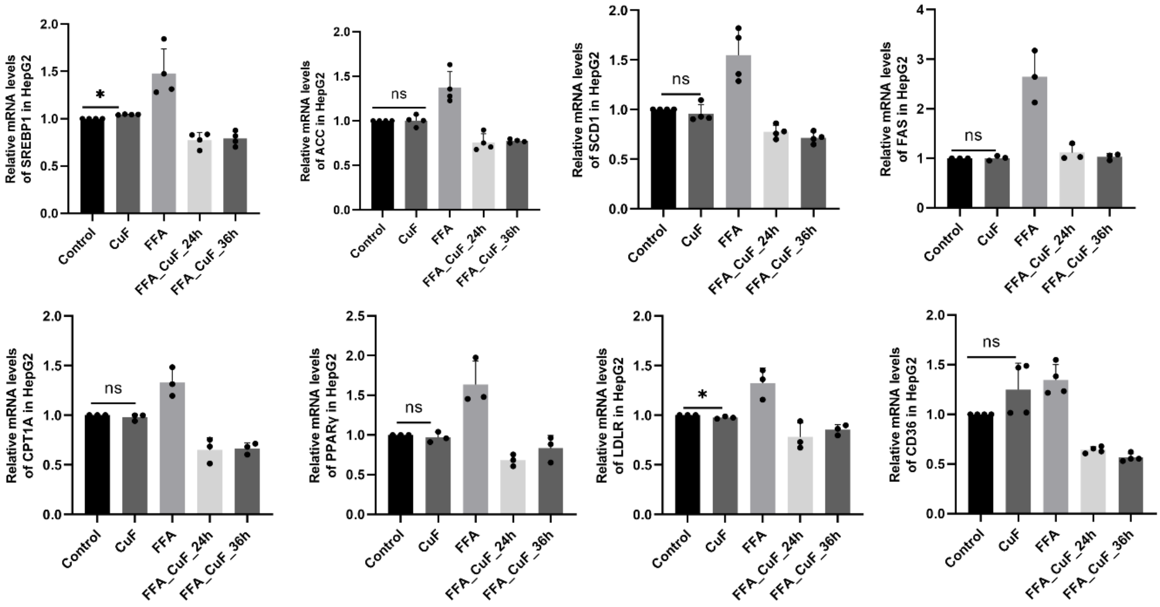

Figure S5. Effects of CuF alone and CuF combined with FFA on fatty acid metabolism–related gene expression in HepG2 cells. (A) Relative mRNA expression of lipogenic genes (SREBP-1c, ACC1, SCD1, and FAS) in HepG2 cells treated with CuF alone and in FFA + CuF co-treatment groups. (B) Relative mRNA expression of fatty acid oxidation–related genes (CPT1A) and lipid uptake–related genes (PPARγ, LDLR, and CD36) following CuF alone or FFA + CuF treatment. CuF alone produced gene expression profiles comparable to the untreated control across all examined pathways, indicating that CuF does not alter basal fatty acid synthesis, oxidation, or lipid uptake. In contrast, CuF co-treatment with FFA significantly modulated these metabolic genes, consistent with the protective effects observed in Figure 4. Data are presented as mean ± SD (n ≥ 3). ns, not significant versus control group; * $p \leq 0.05$ versus control group.

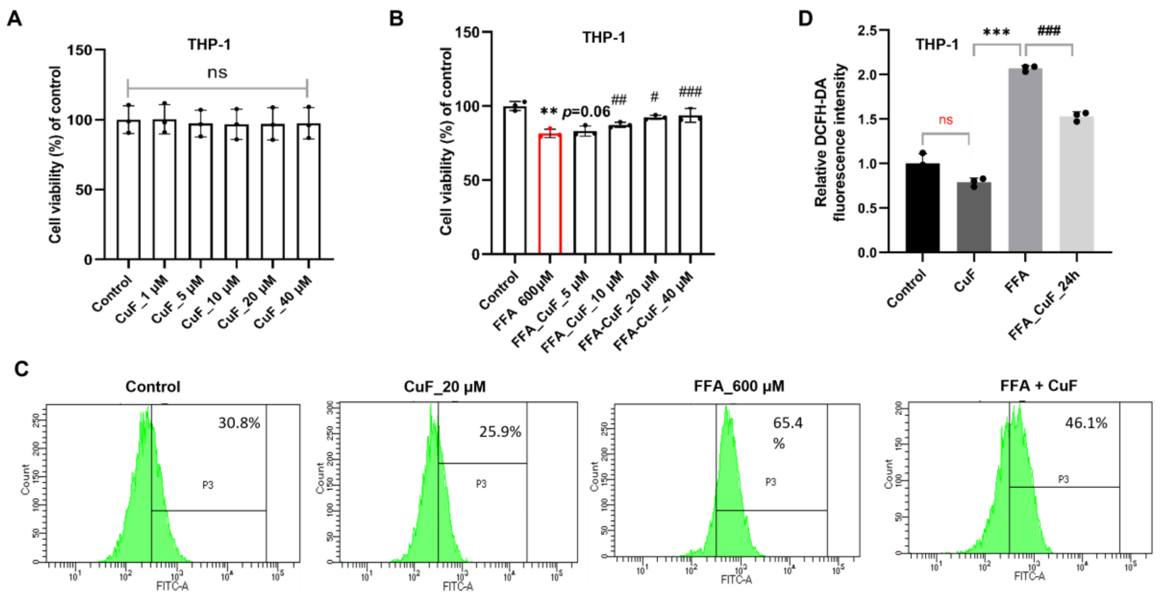

Figure S6. CuF attenuates FFA-induced oxidative stress in THP-1 cells. (A) Cell viability of THP-1 cells treated with CuF alone (0–40 µM) for 36 h. CuF did not cause cytotoxicity at any tested concentration. **(B)** Co-treatment with CuF restored viability in THP-1 cells exposed to 600 µM FFA. Increasing CuF concentrations progressively alleviated FFA-induced viability loss, with significant improvement at 10–40 µM. (C) Representative flow cytometry histograms of intracellular ROS levels (FITC channel) in THP-1 cells. Percentages indicate the proportion of ROS-positive cells within the P3 gate. Treatment with 20 µM CuF for 24h substantially reduced the FFA-induced increase in ROS. (D) Quantification of DCF fluorescence intensity showing intracellular ROS levels under different treatments. CuF alone exhibited ROS levels comparable to the control group. In contrast, FFA markedly increased ROS production, whereas co-treatment with 20 µM CuF effectively attenuated this elevation. Data are presented as mean ± SD (n = 3). ns: not significant; ** $p < 0.01$; *** $p < 0.001$ versus control. # $p \leq 0.05$, ## $p \leq 0.01$, ### $p \leq 0.001$ versus FFA group.

Table S2. Residues contributing to CuF binding identified by MM/PBSA analysis.

Residue	Energy
**Arg415**	**-3.35 kcal/mol**
**Gln556**	**-1.65 kcal/mol**
**Gly509**	**-1.07 kcal/mol**
**Ala510**	**-0.81 kcal/mol**
**Tyr525**	**-0.78 kcal/mol**
**Gly462**	**-0.77 kcal/mol**
**Gly603**	**-0.75 kcal/mol**
**Leu557**	**-0.63 kcal/mol**
**Ile461**	**-0.53 kcal/mol**
**Ser508**	**-0.49 kcal/mol**
**Gly364**	**-0.43 kcal/mol**
**Arg483**	**-0.36 kcal/mol**
**Ser363**	**-0.28 kcal/mol**
**Phe478**	**-0.17 kcal/mol**

Legend: Energies of individual residues calculated using MM/PBSA analysis. Negative values indicate favorable contributions to CuF binding. Energy values are reported in kcal/mol.
